# Association of nursery and early school attendance with later health behaviours, biomedical risk factors, and mortality: evidence from four decades of follow-up of participants in the 1958 birth cohort study

**DOI:** 10.1136/jech-2018-210667

**Published:** 2018-03-14

**Authors:** G David Batty, George B Ploubidis, Alissa Goodman, David Bann

**Affiliations:** 1 Department of Epidemiology and Public Health, University College London, London, UK; 2 Centre for Longitudinal Studies, University College London Institute of Education, London, UK

**Keywords:** cohort studies, education, mortality, social factors in

## Abstract

**Background:**

Although early life education for improved long-term health and the amelioration of socioeconomically generated inequalities in chronic disease is advocated in influential policy statements, the evidence base is very modest.

**Aims:**

To address this dearth of evidence using data from a representative UK national birth cohort study.

**Methods:**

The analytical sample comprised men and women in the 1958 birth cohort study with prospectively gathered data on attendance at nursery or primary school before the age of 5 years who had gone on to participate in social survey at 42 years (n=11 374), or a biomedical survey at 44/5 years of age (n=9210), or had data on vital status from 18 to 55 years (n=17 657).

**Results:**

Relative to study members who had not attended nursery, in those who had, there was in fact a higher prevalence of smoking and high alcohol intake in middle age. Conversely, nursery attenders had more favourable levels of lung function and systolic blood pressure in middle age. This apparent association between nursery attendance and lower systolic blood pressure was confined to study members from more deprived social backgrounds of origin (P value for interaction 0.030). There was no apparent link between early school attendance and any behavioural or biological risk factor. Neither nursery nor early school attendance was clearly related to mortality risk.

**Conclusions:**

We found no clear evidence for an association of either attendance at nursery or primary school before the age of 5 years and health outcomes around four decades later.

## Introduction

Influential policy statements advocate governmental investment in preschool education for long-term improvements in health, and for the alleviation of socioeconomically generated inequalities.^1 2^ This assertion appears to be based on the observation that preschool education is linked to later cognitive and socioeconomic advantage, and, to a lesser degree, social adjustment.^3^ With several common chronic diseases, particularly cardiovascular disease, patterned by these characteristics,^4 5^ a link between early life education and adult health is anticipated. The long-term health effect of preschool education has, however, been little tested.

This modest evidence base can be broadly divided into two areas. Reviews of randomised trials or quasi-experimental studies of multifactorial pre-school interventions at varying intensities of delivery and duration suggest a favourable effect on health behaviours and possibly psychological health.^6^ Additionally, recently, two of the most well-known socio-educational interventions among economically disadvantaged American school children have been revitalised. The Perry Preschool Project took place in the mid-1960s near Michigan (USA), and, one decade later, The Carolina Abecedarian Project was operated from the North Carolina University campus. The intervention arm of these trials had a blood pressure-lowering effect (Abecedarian only) and a favourable impact on health behaviours, most notably smoking,^7 8^ when participants were aged 34–40 years. Effects were typically stronger among male trial members. While these studies have been carefully conducted and study members have, rarely for this field, extensive phenotyping at follow-up, interpretation of the results is hampered by very low sample sizes, leading to modest statistical power, and the relatively short-term health surveillance in the majority of studies.^6^ The location of most of the studies in the USA and the implementation of specialised interventions in socially disadvantaged groups also raises concerns about the generalisability of such finding to the wider population.

Investigators have also used data from observational studies in order to explore the relation of more typical preschool programmes with later health outcomes in the general population. In a recent systematic review, 11 of 12 retrieved studies used self-report health endpoint data which lack utility for those chronic disease outcome that may be asymptomatic such as diabetes and hypertension.^6^ Most studies have yielded null findings, and in the only observational study with objectively measured outcomes of which we are aware,^9^ study members were required to recall participating in a preschool education programme at around 4 years of age some six decades later, leading to concerns about the validity of such a distantly recalled exposure.

In light of the methodological shortcomings of this evidence base, we used data from the National Child Development Study (NCDS)—also known as the 1958 birth cohort study—a well-characterised general population-based birth cohort study with prospective gathered information on attendance at preschool programmes, together with biomedical cardiovascular disease risk factor data. Additionally, for the first time to our knowledge, we explore the association of early educational opportunities with mortality.

## Methods

Described in detail elsewhere,^10^ the NCDS is an ongoing prospective cohort study initially comprising 17 415 births to mothers residing in Great Britain in 1 week of 1958. Following the initial perinatal survey, to date, there have been 10 attempts to contact study members to monitor their physical, educational and social development up to age 55 years. While the contacts with study members as adults have typically been in the context of a social survey with the addition of some physical examinations, exceptionally, at age 44/45 years in 2002/2003, biomedical data were collected when study members took part in a home-based medical examination and survey. Despite inevitable attrition owing to death and refusal to participate, responders to the biomedical sweep remain broadly representative of the original sample.^11^ The study participants provided informed consent and data collection was conducted in accordance with relevant guidelines and regulations.

### Assessment of nursery and preschool attendance

When the participants were aged 7 years, their parents were asked to report the age at which the child began attending school on a full-time basis. This was categorised as early (premandatory: <5 years) or normal/late (≥5 years). Parents were also asked to report whether children attended nursery or preschool classes (local authority or privately funded). These data were categorised as binary variables to indicate attendance status.

### Assessment of biomedical and self-reported cardiovascular disease risk factors

Cardiovascular disease biomarkers were captured at around age 45 years from measurements taken by nurses using standardised protocols. We selected those biomarkers which have either a known relationship with cardiovascular disease events (body mass index, blood pressure, cholesterol and its fractions (high-density lipoprotein (HDL); low-density lipoprotein (LDL)), glycosylated haemoglobin (HbA1c), and forced expiratory volume in 1 s (FEV1),^12^ or an emerging one (triglycerides; fibrinogen; C-reactive protein (CRP); D-dimer; tissue plasminogen activator (tPA); and von Willebrand factor (vWF)).^13 14^

After participants had been seated for 5 min, blood pressure was measured three times (Omron 705CP, Tokyo, Japan) with mean values for each component used in the present analyses. Venous blood samples were obtained without prior fasting and posted to collaborating laboratories. From these, HbA1c levels were measured using ion exchange high performance liquid chromatography; fibrinogen was determined by the Clauss method using a MDA 180 coagulometer; CRP was assayed by nephelometry (Dade Behring) on citrated plasma samples after one thaw cycle; vWF antigen was measured by Decollates ELISA and tPA antigen (Biopool, Sweden) and total cholesterol, HDL-cholesterol and triglyceride levels were analysed using an autoanalyzer (Olympus AU640, Japan) using enzymatic methods. LDL-cholesterol levels were calculated using the Friedewald formula (total cholesterol–(HDL+[Trig/2.2])). Fibrin D-dimer was measured on stored samples at the end of the field study period by ELISA (Hyphen, Paris, France) and standardised for interbatch variation.

Body mass index (weight, kg/(height)^2^ m^2^) was calculated using height (Leicester portable stadiometers) and weight (Tanita solar scales) measurements taken using standardised protocols with participants wearing light clothing and having removed their shoes. In the standing position, without nose clips and using the Vitalograph Mirco spirometer (Vitalograph, Maids Moreton, UK), at least three (maximum five) spirograms were captured until three satisfactory results were obtained. The highest technically satisfactory value for FEV1 was used. Behavioural risk factors were assessed at the age 42 follow-up when self-reported data were gathered on cigarette smoking (for the purposes of analyses, categorised as none vs current smoker), leisure time exercise participation (<1/month vs more) and intake of fruits, vegetables (<1/week vs more) and alcohol consumption (most days vs fewer).

### Assessment of confounding factors

The following potential confounders were self-reported by the mother of the study member either at birth or when the study member was 7 years of age. At birth, enquiries were made regarding paternal occupational class (six categories from professional to unskilled), maternal attendance in postcompulsory school (yes/no), overcrowding (1, 1–2 or >2 people/room), self-reported maternal weight (in 10 categories from <7 to >15 stone (1 stone is 14 pounds or 6.35 kg)) and smoking status (non-smoker, and 6 categories of amount from 1 to 4 to ≥30 cigarettes daily). When the study member was 7 years of age, mothers also responded to questions about her interest in the child’s education (overly concerned, very, some or little interest) and whether the child had been breast fed (yes or no); a positive response to the latter led to a follow-up enquiry about duration (<1 or >1 month).

### Ascertainment of mortality

Vital status was ascertained up to 55 years of age (December 2013) using death certificates supplied by the National Health Service Central Register and/or notification given by family members of participants. All-cause mortality from 18 up to age 55 years was used in analyses.

### Statistical analyses

We examined associations between early life education and potential confounders using Χ^2^ tests for heterogeneity across education groups. We evaluated the relationship between early life education experience and adult biomedical risk factors for cardiovascular disease, all continuous variables, using linear regression analyses in which we computed beta-coefficients with accompanying 95% CIs. To aid the comparability of coefficients, outcomes were standardised and, in some cases, log-transformed to normalise their distribution prior to conversion (HbA1c, HDL, triglycerides, fibrinogen, CRP, D-dimer, tPA and vWF). Having first determined that the proportional hazard assumption had not been violated, we computed hazard ratios (95% CI) using Cox regression models to evaluate the relationship between early life education and total mortality.

This being a birth cohort study, no adjustment for age was made in these analyses. Also, with no modification by gender apparent in preliminary analyses, effect estimates were first gender-adjusted, with the remaining confounding factors then added to the regression model. To maintain power and minimise potential bias resulting from missing data, full information maximum likelihood estimation was used to account for missing exposure and confounder data, and multiple imputation was used for Cox regression analyses (with 10 imputed datasets). These methods yield unbiased results under the missing at-random assumption.^15^

To examine whether higher-resourced early education and/or differences in access to such resources was related to the health outcomes herein, we repeated the above analyses using a binary indicator for private (fee-paying) nursery attendance. As most trial evidence in this area comes from the recruitment of families from socioeconomically deprived communities,^6^ and health benefits may be more pronounced among socioeconomically disadvantaged subgroups, we also tested for effect modification by paternal social class, presenting stratified analyses to illustrate any such interaction.

## Results

In table 1, we show study member characteristics according to categories of nursery and early school education. Around 18% of study members attended nursery and around 51% attended school before the age of 5 years. Of those study members who attended nursery, 60% subsequently had experience of early schooling. Study members who attended nursery were advantaged relative to those that did not: they were less likely to come from poorer social circumstances—indexed by paternal social class, mother’s educational experience and overcrowding at home—and have a mother uninterested in their offspring’s education. Socioeconomic differentials were also evident in later life, whereby individuals exposed to nursery were less likely to be in a manual occupation by middle age. The maternal health behaviours of such study members who attended nursery were also more favourable, such that mothers were less likely to have smoked during pregnancy and to have breast fed the study member. While this pattern of association was also apparent for early school attendance, the differentials were typically less pronounced than for nursery attendance. Potential confounders were generally correlated with each other in expected directions, for example, higher paternal occupational class was correlated with higher maternal education attainment, although the magnitude of the relationships was low to moderate (results not shown).

**Table 1:** Study member characteristics according to nursery and early school attendance

	No nursery	Nursery attended	P value for difference		Normal-late school attendance (≥5 years)	Early school attendance (<5 years)	P value for difference
Total sample	10 523 (82.4)	2241 (17.6)			7104 (49.3)	7296 (50.6)	
Male	5368 (51.0)	1135 (50.6)	<0.8		3693 (52.0)	3692 (50.6)	0.1
Manual father’s social class	7303 (75.0)	1294 (64.3)	<0.001		4720 (72.7)	4938 (73.0)	<0.01
Non-attendance of mother at postcompulsory school	7933 (78.1)	1418 (66.4)	<0.001		5237 (76.9)	5206 (73.8)	<0.01
Overcrowding in the home (>1 person/room)	3281 (33.0)	558 (26.8)	<0.001		2337 (35.0)	1916 (27.8)	<0.01
Mother little interested in child’s education	1524 (16.0)	286 (14.3)	<0.001		1118 (17.6)	905 (13.6)	<0.01
Mother smoked prior to pregnancy	3874 (39.1)	782 (37.9)	0.21		2635 (39.8)	2608 (37.9)	0.03
Mother did not breast feed	3402 (32.6)	604 (27.2)	<0.001		2323 (33.1)	2186 (30.2)	<0.01
Mother’s weight >10 stones	2814 (28.3)	555 (26.5)	0.16		1868 (28.0)	1935 (27.9)	0.5
Own manual occupational social class (42 years)	2278 (37.4)	361 (28.8)	<0.001		1493 (37.5)	1474 (34.1)	<0.01

Results are N (%).

In table 2, we show odds ratios for the relation of nursery and early school attendance at 5 years of age with self-reported behavioural cardiovascular disease risk factors at age 42 years. After adjustment for multiple covariates, study members who attended nursery relative to those that did not had an elevated prevalence of adult smoking and high alcohol intake. Attending school early was largely unrelated to any of the four health behaviours in adulthood.

**Table 2:** Odds ratios (95% CI) for the relation of nursery and early school attendance with self-reported health-related behavioural outcomes at age 42 years

	N	No nursery	Nursery attended	Normal-late school attendance (≥5 years)	Early school attendance (<5 years)
**Sex-adjusted**					
Current smoker	11 374	Ref.	1.13 (1.00 to 1.27)	Ref.	0.91 (0.83 to 0.99)
Low exercise frequency (<1/month)	11 372	–	1.00 (0.89 to 1.12)	–	0.95 (0.88 to 1.04)
Low fruit and vegetable/salad intake (<1/week)	11 371	–	0.81 (0.69 to 0.94)	–	0.96 (0.86 to 1.06)
High alcohol consumption frequency (most days)	11 374	–	1.24 (1.08 to 1.42)	–	1.13 (1.02 to 1.24)
**Multiply-adjusted**					
Current smoker	11 374	–	1.28 (1.11 to 1.48)	–	0.99 (0.89 to 1.09)
Low exercise frequency (<1/month)	11 372	–	1.09 (0.95 to 1.25)	–	1.01 (0.91 to 1.11)
Low fruit and vegetable/salad intake (<1/week)	11 371	–	0.93 (0.78 to 1.11)	–	0.97 (0.86 to 1.10)
High alcohol consumption frequency (most days)	11 374	–	1.21 (1.03 to 1.41)	–	1.04 (0.93 to 1.17)

Multiple-adjustment is adjustment for potential confounders measured at birth (father’s social class, mother’s attendance at postcompulsory school, overcrowding in the home, mother’s smoking status prior to pregnancy, mother’s weight) and 7 years (maternal interest in the child’s education, breast feeding).

In table 3, we summarise the relation of nursery and early school attendance with biomedical risk factors for cardiovascular disease at age 44/45 years. Of the 14 risk factor outcomes, in the basic analyses where associations were adjusted for sex, there were few associations apparent. Nursery attendance was associated with more favourable lung function and systolic blood pressure. With addition of further covariates to the multivariable model, the magnitude of these associations were somewhat diminished and statistical significance at conventional levels lost. Attenuation appeared to have been driven by the inclusion of parental socioeconomic factors. For example, after control for father’s occupational class, maternal education and overcrowding, mean difference in systolic blood pressure among those who attended nursery relative to those who did not (beta-coefficient; 95% CI −0.05; −0.11 to 0.01) the same as that apparent for the fully adjusted results (−0.05; −0.11 to 0.01). There was no apparent link between early school attendance and any of the biological end points. When we examined if nursery attendance or early education was related to all-cause mortality up to 55 years of age, we found no such suggestion (table 4).

**Table 3:** Beta-coefficients (95% CI) for the relation of nursery and early school attendance with an SD increase in biomedical outcomes at age 44/45 years

	Mean (SD)	N	No nursery	Nursery attended	Normal-late school attendance (≥5 years)	Early school attendance (<5 years)
**Sex-adjusted**						
Body mass index, kg/m^2^	27.4 (5.0)	9210	Ref.	0.01 (-0.05 to 0.07)	Ref.	0.00 (−0.04 to 0.05)
Systolic blood pressure, mm Hg	127.2 (16.8)	9257	–	−0.07 (-0.12 to–0.01)	–	0.03 (−0.01 to 0.07)
Diastolic blood pressure, mm Hg	79.2 (10.9)	9257	–	−0.05 (− 0.11 to 0.01)	–	0.02 (− 0.02 to 0.06)
HbA1c, %	1.7 (0.1)	7923	–	−0.04 (− 0.11 to 0.02)	–	−0.01 (− 0.06 to 0.04)
FEV1, L	3.2 (0.9)	9091	–	0.06 (0.01 to 0.11)	–	0.04 (0.00 to 0.08)
Total cholesterol, mmol/L	5.9 (1.1)	7824	–	0.03 (− 0.04 to 0.10)	–	0.03 (−0.01 to 0.08)
HDL, mmol/L	0.4 (0.2)	7808	–	0.03 (− 0.04 to 0.09)	–	0.02 (− 0.03 to 0.06)
LDL, mmol/L	3.4 (0.9)	7391	–	0.05 (− 0.02 to 0.12)	–	0.03 (− 0.02 to 0.08)
Triglycerides, mmol/L	0.5 (0.6)	7799	–	−0.02 (− 0.08 to 0.04)	–	0.03 (− 0.02 to 0.07)
Fibrinogen, g/L	1.1 (0.2)	7683	–	−0.06 (− 0.13 to 0.01)	–	−0.04 (− 0.08 to 0.01)
CRP, g/L	0.0 (1.2)	7692	–	−0.06 (− 0.13 to 0.01)	–	0.02 (− 0.03 to 0.06)
D-dimer, ng/mL	5.1 (0.6)	7651	–	0.00 (− 0.07 to 0.07)	–	−0.01 (− 0.05 to 0.04)
tPA, ng/mL	1.5 (0.6)	7692	–	−0.06 (− 0.12 to 0.01)	–	0.01 (− 0.04 to 0.05)
vWF, IU/dL	4.8 (0.3)	7693	–	−0.03 (− 0.10 to 0.04)	–	−0.02 (− 0.07 to 0.03)
**Multiply-adjusted**						
Body mass index, kg/m^2^	–	9210	–	0.06 (0.00 to 0.13)	–	0.02 (− 0.02 to 0.06)
Systolic blood pressure, mm Hg	–	9257	–	−0.05 (− 0.11 to 0.01)	–	0.03 (− 0.01 to 0.07)
Diastolic blood pressure, mm Hg	–	9257	–	−0.03 (− 0.09 to 0.03)	–	0.02 (− 0.02 to 0.07)
HbA1c, %	–	7923	–	−0.01 (− 0.08 to 0.05)	–	0.00 (− 0.05 to 0.05)
FEV1, L	–	9091	–	0.03 (− 0.02 to 0.09)	–	0.02 (− 0.01 to 0.06)
Total cholesterol, mmol/L	–	7824	–	0.04 (− 0.03 to 0.11)	–	0.04 (− 0.01 to 0.08)
HDL, mmol/L	–	7808	–	−0.01 (− 0.08 to 0.05)	–	0.00 (− 0.04 to 0.05)
LDL, mmol/L	–	7391	–	0.07 (0.00 to 0.14)	–	0.03 (− 0.01 to 0.08)
Triglycerides, mmol/L	–	7799	–	0.02 (− 0.05 to 0.08)	–	0.04 (0.00 to 0.09)
Fibrinogen, g/L	–	7683	–	−0.02 (− 0.08 to 0.05)	–	−0.02 (− 0.07 to 0.03)
CRP, g/L	–	7692	–	−0.01 (− 0.08 to 0.06)	–	0.04 (− 0.01 to 0.08)
D-dimer, ng/mL	–	7651	–	0.02 (− 0.04 to 0.09)	–	0.00 (− 0.04 to 0.05)
tPA, ng/mL	–	7692	–	−0.04 (− 0.10 to 0.03)	–	0.02 (− 0.03 to 0.06)
vWF, IU/dL	–	7693	–	−0.01 (− 0.07 to 0.06)	–	−0.01 (− 0.06 to 0.04)

Multiple-adjustment is adjustment for potential confounders measured at birth (father’s social class, mother’s attendance at postcompulsory school, overcrowding in the home, mother’s smoking status prior to pregnancy, mother’s weight) and 7 years (maternal interest in the child’s education, breast feeding).

CRP, C reactive protein; FEV1, forced expiratory volume in 1 s; HbA1c, glycosylated haemoglobin; HDL, high-density lipoprotein; LDL, low-density lipoprotein; tPA, tissue plasminogen activator; vWF, von Willebrand factor.

**Table 4:** Hazrads ratios (95% CI) for the relation of nursery and early school attendance with all-cause mortality up to 55 years (n=17 657)

	No nursery	Nursery attended	Normal-late school attendance (≥5 years)	Early school attendance (<5 years)
Number deaths	485	86	325	317
Sex-adjusted	1.0 (Ref.)	0.82 (0.67 to 1.02)	1.0 (Ref.)	1.02 (0.86 to 1.20)
Multiply adjusted	1.0	0.89 (0.69 to 1.17)	1.0	1.04 (0.89 to 1.23)

Multiple-adjustment is adjustment for potential confounders measured at birth (father’s social class, mother’s attendance at postcompulsory school, overcrowding in the home, mother’s smoking status prior to pregnancy, mother’s weight) and 7 years (maternal interest in the child’s education, breast feeding).

While the prevalence of attending private nursery was low (5.8%), as evident in online supplementary table 1, associations between private nursery attendance and outcomes were more frequent and of greater magnitude than in the previously described analyses for any nursery attendance. This was particularly evident in the most basic model where children who attended a private nursery/class, relative to those that did not, had more favourable biomedical markers levels, including body mass index, both components of blood pressure, HbA1c, lung function, HDL, triglycerides, fibrinogen, CRP, D-dimer, tPA and vWF. The magnitude of all these relationships was, however, heavily attenuated after adjustment for multiple covariates which included socioeconomic position, and only the confidence intervals for FEV1 and tPA did not include zero. Private nursery attendance was not, however, related to adult all-cause mortality after statistical adjustment (multiply adjusted HR; 95% CI 1.02; 0.69 to 1.52). Similar results were apparent when deaths from childhood were included in the total mortality outcome.

10.1136/jech-2018-210667.supp1 Supplementary file 1

As apparent in online supplementary table 2, there was little evidence that the relations between the two indicators of early education and later outcomes differed systematically by socioeconomic background, as indexed by father’s occupational class. The only consistent exception was the suggestion of a differential effect for the association between any nursery attendance and blood pressure in adulthood where protective effects were apparent among those with fathers of lower (manual) occupational class only (P value for interaction: 0.03 for systolic blood pressure and 0.06 for diastolic blood pressure). When total mortality was the outcome of interest, there was no suggestion of effect modification by paternal social class (P value for interaction was 0.2 for nursery and 0.08 for early school attendance). Lastly, thus far in our analyses we have used the maximum sample size for each statistical model. As discussed, while this has the advantage of maximising statistical power and minimising bias, results across different models are not strictly comparable. We therefore conducted additional analyses by using the same subsample with valid data for all outcomes (ie, a so-called ‘non-missing’ dataset). The results did not substantially differ.

## Discussion

In our main analyses of data from this prospective birth cohort study, there was little consistent evidence that early education or nursery attendance were associated with cardiovascular disease risk factors and mortality in middle age, after accounting for early life potential confounding factors such as parental socioeconomic status. While adult smoking and high alcohol intake were in fact more common in cohort members who, as children, had attended nursery, favourable levels of lung function and systolic blood pressure were also apparent in this group. When stratifying analyses according to early life social circumstances in an effort to mimic the samples of several trials of intensive educational interventions which have largely focused on socioeconomically disadvantaged groups,^7^ this apparent association between nursery attendance and lower blood pressure in adults was confined to study members from more deprived social backgrounds. The most unambiguous set of results was apparent for private (fee-paying) nursery attendance, which seemed to be associated with more favourable levels of a range of biological risk factors, although these were largely eliminated after adjustment for early life socioeconomic status.

### Evidence from existing studies

With existing studies being based on multiple study designs (cross-sectional studies, cohort studies, trials) and with interventions that also differ markedly in content, intensity of delivery, and population recruited, contextualising our findings is not straightforward. In the only observational study with biological end points of which we are aware,^9^ as indicated, study members were required to recall participating in a preschool education programme at around 4 years of age some six decades later. Notwithstanding the concerns about the validity of such a distantly recalled exposure, there was little evidence of an association with multiple cardiovascular disease-related outcomes.^9^ In studies in which the investigators relied exclusively on self-reported hypertension—the Perry Preschool^16^ and the Head Start^17^ trials—the prevalence was actually elevated in the group experiencing more intensive education. In contrast, in a follow-up of participants in the Carolina Abecedarian Project in which the focus was cognitive and social stimulation from birth to 5 years, there was a markedly lower blood pressure level in the intervention arm relative to the non-treatment control group.^7^

### Study strengths and limitations

Relative to trial evidence where, as discussed, samples are typically drawn from deprived communities, and where the educational interventions are generally intensive, data from the present study offer insights into the effectiveness in a population-wide (‘real-world’) context, although using data from an observational study has its attendant limitations. We were, however, also able to examine links with a wider array of cardiovascular disease risk factors than has previously been possible and, for the first time to our knowledge, explore associations with mortality.

Our work is of course not without its shortcomings. Our measure of early school and nursery attendance was not detailed, failing, for instance, to capture the content of the experience and the reason for such attendance. This notwithstanding, these data appear to have some predictive utility, being related to markers of social circumstances both concurrently and prospectively.^3^ We tested the association of early educational systems and nursery practices that prevailed 50 years ago; the extent to which these have relevance for current circumstances is uncertain, as are the alternatives (eg, home parenting provision given increasing labour market participation of women). It is also the case that the extent to which skills provided to children in preschool in the era of data collection of the present study that may have been useful for subsequent life skills or educational pathways is likely to have evolved. These are, however, necessary features of any study which aims to explore the long-term health effects of an early life exposure into adulthood. There is some evidence to suggest that the positive impacts of educational interventions, seen, for instance, for cognitive function in adolescents, subsequently dissipate.^18^ This diminution of effect with time could be the same for cardiovascular disease risk factors as outcomes but with these data not having been ascertained earlier in the life course in the present study precludes any such exploration. Related, there may have been advantages for current study members who received early schooling or nursery in health domains that we did not investigate, such as mental health. Additionally, other early life educational-type characteristics were not collected in the present study such as alternative child care arrangements. Our health behaviour outcomes were all self-reported which may be regarded as a weakness. However, to the best of our knowledge, other than for physical activity (accelerometry, which is costly) and smoking (cotinine, which has a half-life short enough to raise concerns about its ability to quantify usual exposure), there are currently no biomarkers of alcohol, or fruit and vegetable intake of acceptable utility for population-based research when many thousands of people are surveyed. Given the very large number of statistical tests necessarily conducted in the course of our analyses, our few positive results may be chance findings. Lastly, our results are based on observational data, so confounding by known and unknown factors is a perennial if rather hackneyed concern. With the impact of confounding typically being attenuation of exposure–outcome relationships, and with most of our results being null, this is unlikely to be an issue in the present study, however.

In conclusion, we found little consistent evidence of an association of early life education and nursery attendance with adult risk factors and total mortality. Future studies are needed to examine specific types of educational programmes where content and quality is captured in more detail.

**What is already known on this subject**

- The long-term health effect of preschool education has been little tested.
- Interpretation of trial evidence is hampered by very low sample sizes and the relatively short-term health surveillance in the majority of studies.
- Observational studies typically use self-reported health outcome data and the distant recall of early life education raises concerns regarding validity.

**What this study adds**

- To the best of our knowledge, this is the first observational study with prospective data on early educational experience and objectively measured health outcomes.
