# Supplementary material for: Association of nursery and early school attendance with later health behaviours, biomedical risk factors, and mortality: evidence from four decades of follow-up of participants in the 1958 birth cohort study
Source: J Epidemiol Community Health. 2018 Mar 14;72(7):658–63. doi: 10.1136/jech-2018-210667 (PMC6031276; doi:10.1136/jech-2018-210667)
Supplement: Supplementary file 1 [file jech-2018-210667supp001.pdf]

Supplemental Table 1. Private nursery/class attendance (for at least 1 month) relative to none in relation to a standard deviation increase in health-related outcomes at age 44/45 years

	N	Did not attend (Ref.)	Attended private nursery or class β (95% CI)
**Sex-adjusted**			
Body mass index, kg/m^2	9210	-	-0.21 (-0.30, -0.11)
Systolic blood pressure, mmHg	9257	-	-0.14 (-0.23, -0.05)
Diastolic blood pressure, mmHg	9257	-	-0.13 (-0.22, -0.04)
HbA1c, %	7923	-	-0.15 (-0.25, -0.05)
FEV1, l	9091	-	0.19 (0.11, 0.26)
Cholesterol, mmol/l	7824	-	0.00 (-0.10, 0.10)
HDL, mmol/l	7808	-	0.22 (0.12, 0.32)
LDL, mmol/l	7391	-	0.02 (-0.09, 0.12)
Triglycerides, mmol/l	7799	-	-0.22 (-0.31, -0.12)
Fibrinogen, g/l	7683	-	-0.21 (-0.32, -0.11)
CRP, g/l	7692	-	-0.27 (-0.38, -0.17)
D-dimer, ng/ml	7651	-	-0.11 (-0.21, -0.01)
tPA, ng/ml	7692	-	-0.21 (-0.31, -0.11)
VWF, IU/dl	7693	-	-0.12 (-0.22, -0.01)
**Multiply-adjusted**			
Body mass index, kg/m^2	9210	-	-0.04 (-0.14, 0.06)
Systolic blood pressure, mmHg	9257	-	-0.06 (-0.15, 0.03)
Diastolic blood pressure, mmHg	9257	-	-0.06 (-0.15, 0.04)
HbA1c, %	7923	-	-0.06 (-0.17, 0.05)
FEV1, l	9091	-	0.08 (0.00, 0.17)
Cholesterol, mmol/l	7824	-	0.04 (-0.07, 0.14)
HDL, mmol/l	7808	-	0.07 (-0.03, 0.18)
LDL, mmol/l	7391	-	0.07 (-0.03, 0.18)
Triglycerides, mmol/l	7799	-	-0.09 (-0.19, 0.01)
Fibrinogen, g/l	7683	-	-0.05 (-0.16, 0.06)
CRP, g/l	7692	-	-0.10 (-0.20, 0.01)
D-dimer, ng/ml	7651	-	-0.03 (-0.14, 0.07)
tPA, ng/ml	7692	-	-0.13 (-0.23, -0.02)
VWF, IU/dl	7693	-	-0.02 (-0.13, 0.09)

Multiple-adjustment is adjustment for potential confounders measured at birth (parental occupational class, maternal attendance in post compulsory school, overcrowding in the home, maternal weight and smoking status) or 7 years (maternal interest in the child's education and breastfeeding).

Supplemental Table 2. Multiple-adjusted beta coefficients (95% confidence interval) for the relation of nursery and early school attendance with a standard deviation increase biomedical outcomes at age 44/45 years according to father's occupational class at birth

	N	No nursery	Nursery attended	P-value for interaction		Normal-late school attendance (≥5 years)	Early school attendance (<5 years)	P-value for interaction*
**Higher father's occupational class at birth (class I to IIIm):**								
Body mass index, kg/m^2	6645	Ref.	0.02 (-0.05, 0.09)	0.453		Ref.	0.01 (-0.04, 0.06)	0.752
Systolic blood pressure, mmHg	6681	-	0.00 (-0.06, 0.07)	0.030		-	0.04 (0.00, 0.09)	0.008
Diastolic blood pressure, mmHg	6681	-	0.02 (-0.05, 0.09)	0.059		-	0.03 (-0.02, 0.08)	0.085
Glycosylated haemoglobin, %	5727	-	0.00 (-0.07, 0.07)	0.415		-	-0.02 (-0.07, 0.03)	0.489
FEV1, l	6562	-	0.05 (-0.01, 0.11)	0.410		-	0.04 (-0.01, 0.08)	0.824
Total cholesterol, mmol/l	5649	-	0.07 (0.00, 0.15)	0.540		-	0.04 (-0.01, 0.10)	0.001
HDL, mmol/l	5639	-	0.03 (-0.05, 0.10)	0.463		-	0.00 (-0.06, 0.05)	0.461
LDL, mmol/l	5357	-	0.09 (0.02, 0.17)	0.762		-	0.04 (-0.02, 0.10)	0.008
Triglycerides, mmol/l	5631	-	0.00 (-0.07, 0.08)	0.362		-	0.05 (0.00, 0.10)	0.307
Fibrinogen, g/l	5564	-	-0.05 (-0.13, 0.03)	0.995		-	-0.05 (-0.10, 0.01)	0.251
CRP, g/l	5570	-	-0.05 (-0.13, 0.03)	0.777		-	0.02 (-0.03, 0.08)	0.170
D-dimer, ng/ml	5539	-	0.02 (-0.06, 0.10)	0.611		-	-0.02 (-0.07, 0.04)	0.300
tPA, ng/ml	5570	-	-0.05 (-0.13, 0.02)	0.291		-	0.01 (-0.05, 0.06)	0.287
VWF, IU/dl	5570	-	-0.06 (-0.14, 0.02)	0.343		-	-0.01 (-0.06, 0.05)	0.945
**Lower father's occupational class at birth (class IV and V):**								
Body mass index, kg/m^2	1676	Ref	0.08 (-0.09, 0.25)			Ref.	-0.02 (-0.13, 0.08)	
Systolic blood pressure, mmHg	1684	-	-0.22 (-0.38, -0.07)			-	-0.05 (-0.15, 0.05)	
Diastolic blood pressure, mmHg	1684	-	-0.21 (-0.36, -0.05)			-	-0.06 (-0.15, 0.04)	
HbA1c, %	1434	-	-0.14 (-0.34, 0.06)			-	0.04 (-0.09, 0.16)	
FEV1, l	1651	-	0.01 (-0.12, 0.14)			-	0.01 (-0.08, 0.09)	
Total cholesterol, mmol/l	1419	-	-0.10 (-0.28, 0.09)			-	0.03 (-0.08, 0.14)	
HDL, mmol/l	1414	-	-0.13 (-0.30, 0.04)			-	0.07 (-0.03, 0.17)	
LDL, mmol/l	1317	-	-0.04 (-0.22, 0.14)			-	0.02 (-0.09, 0.13)	
Triglycerides, mmol/l	1416	-	-0.02 (-0.20, 0.17)			-	-0.02 (-0.13, 0.09)	
Fibrinogen, g/l	1386	-	-0.06 (-0.24, 0.12)			-	0.03 (-0.08, 0.14)	
CRP, g/l	1389	-	0.06 (-0.12, 0.23)			-	0.03 (-0.07, 0.14)	
D-dimer, ng/ml	1384	-	-0.04 (-0.22, 0.14)			-	0.05 (-0.06, 0.15)	
tPA, ng/ml	1389	-	-0.01 (-0.19, 0.16)			-	0.01 (-0.10, 0.12)	
VWF, IU/dl	1390	-	0.10 (-0.07, 0.28)			-	-0.02 (-0.13, 0.09)	

*P-values were calculated using all 6 father's occupational class categories, and effect estimates presented in binary format to aid presentation. Effect estimates in all 6 groups are shown below (where P<0.1), for I (highest) to V (lowest) occupational class.

Multiple-adjustment is adjustment for potential confounders measured at birth (parental occupational class, maternal attendance in post compulsory school, overcrowding in the home, maternal weight and smoking status) or 7 years (maternal interest in the child's education and breastfeeding).
